# Let-7f-5p suppresses Th17 differentiation via targeting STAT3 in multiple sclerosis

**DOI:** 10.18632/aging.102093

**Published:** 2019-07-15

**Authors:** Zhi-Hui Li, Yi-Fei Wang, Dan-Dan He, Xue-Mei Zhang, Ying-Lian Zhou, Hui Yue, Shan Huang, Zheng Fu, Ling-Yu Zhang, Zhu-Qing Mao, Shuang Li, Chen-Yu Zhang, Xi Chen, Jin Fu

**Affiliations:** 1 Department of Neurology, The Second Affiliated Hospital of Harbin Medical University, Harbin Medical University, Harbin, Heilongjiang 150086, China; 2 Department of Neurology, The Fourth Affiliated Hospital of Harbin Medical University, Harbin Medical University, Harbin, Heilongjiang 150086, China; 3 Jiangsu Engineering Research Center for microRNA Biology and Biotechnology, School of Life Sciences, Nanjing University, Nanjing, Jiangsu 210093, China

**Keywords:** multiple sclerosis, Th17, miRNA, let-7f-5p, STAT3

## Abstract

T helper 17 (Th17) cells are regarded as key factors in the pathogenesis of multiple sclerosis (MS). Although the involvement of certain microRNAs (miRNAs) in the development of MS has been reported, their roles in Th17 cell differentiation and MS pathogenesis remain elusive. In this study, we identified that let-7f-5p expression is significantly downregulated in CD4^+^ T cells from MS patients and during the process of Th17 differentiation. The overexpression of let-7f-5p suppressed Th17 differentiation, whereas the knockdown of let-7f-5p expression enhanced this progress. We then explored the molecular mechanism through which let-7f-5p suppressed Th17 differentiation and identified signal transducer and activator of transcription 3 (STAT3), a pivotal transcription factor of Th17 cells, as a direct target of let-7f-5p. In contrast to the downregulated expression of let-7f-5p, STAT3 and p-STAT3 protein levels were dramatically upregulated and inversely correlated with let-7f-5p in peripheral blood CD4^+^ T cells from MS patients. In conclusion, let-7f-5p functions as a potential inhibitor of Th17 differentiation in the pathogenesis of MS by targeting STAT3 and may serve as a new therapeutic target.

## INTRODUCTION

Multiple sclerosis (MS) is a chronic demyelinating neurodegenerative autoimmune disease of the central nervous system (CNS) and is a major cause of disability in young adults in the developed world [1, 2]. Due to the limited understanding of the pathogenesis of MS and the absence of diagnostic biomarkers, the present diagnostic criteria currently used for MS still primarily rely on clinical manifestations [3]. However, patients with MS may suffer different degrees of motor and sensory dysfunction, which may share considerable similarities with other disorders of the CNS, leading to diagnostic and therapeutic difficulties. Although the precise pathogenesis of MS remains elusive, CD4^+^ T cell-mediated autoimmunity may play an essential role [4]. After antigen stimulation, naive CD4^+^ T cells can activate, proliferate and differentiate into different subsets of T helper (Th) cells, such as Th1, Th2, Th17 and induced regulatory T cells [5, 6].

Th17 cells, an effector subset that secretes IL-17, IL-21 and IL-22, are likely to be the most crucial pathogenic factors of MS and experimental autoimmune encephalomyelitis (EAE) [7, 8]. The differentiation of Th17 cells is a complicated process and regulated by a series of transcription factors, in which retinoic acid receptor-related orphan receptor-γt (RORγt) and signal transducer and activator of transcription 3 (STAT3) play critical roles. Through the activation and phosphorylation of STAT3 and downstream regulation of RORγt [8, 9], naive CD4^+^ T cells differentiate into Th17 cells. The frequencies of Th17 cells have been shown to be upregulated in the peripheral blood and cerebrospinal fluid (CSF) of MS patients and are further elevated during relapses [10]. Mice with fewer Th17 cells are less susceptible to EAE [11], and IL-17-secreting T cells have been observed in the brain lesions of MS patients. The levels of IL-17-secreting T cells are also elevated in the active areas of MS lesions compared with those in the inactive areas [12]. MicroRNAs (miRNAs) are small, conserved, single-stranded noncoding RNA molecules (18-22 nucleotides), that bind to the 3’ untranslated regions (3’-UTRs) of target genes, suppressing their expression and function [13, 14]. Over the past decade, more than 1,000 miRNAs have been identified, and most of which have been shown to regulate various biological activities, including organ development, cellular development and differentiation, signal transduction and immune response [15, 16]. In recent years, many researchers have shown that miRNAs may function in the pathogenesis and progression of MS [17–19]. However, the altered expression and possible role of miRNAs in the Th17 differentiation and pathogenesis of MS remain largely unknown.

In the present study, we revealed that let-7f-5p was consistently downregulated in the peripheral blood CD4^+^ T cells from MS patients. Subsequently, we found that let-7f-5p suppressed the Th17 differentiation without influencing apoptosis. Furthermore, we validated that STAT3 is a direct target gene of let-7f-5p and confirmed that let-7f-5p mediates Th17 differentiation by inhibiting STAT3 expression. Thus, our results suggest that let-7f-5p is a Th17 cell-associated miRNA that functions in the pathogenesis of MS by negatively regulating STAT3.

## RESULTS

### miRNA expression profiling of peripheral blood CD4^+^ T cells from MS patients using Illumina high-throughput sequencing

**Figure 1:**
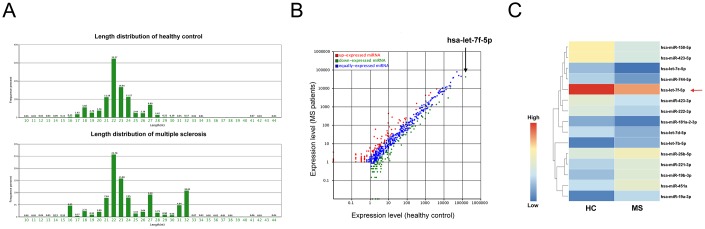
**MiRNA expression profiling of peripheral blood CD4^+^ T cells from MS patients and healthy controls.** (**A**) The lengths of small RNAs from the two pooled samples from the multiple sclerosis (MS) patients and healthy controls (HC). (**B**) Significant alterations were observed between the miRNA expression profiles of the MS patients and healthy controls. (**C**) Heat map of the significantly altered miRNAs (sequencing reads > 200 and fold-change > 2) obtained from Illumina high-throughput sequencing.

High-throughput sequencing was performed for peripheral blood CD4^+^ T cells from MS patients and healthy controls (HC) using total RNA extracted from the cells of individuals from each group (CD4^+^ T cells sorted from ten individuals were pooled). The two samples contained small RNAs with a variety of lengths (Figure 1A), with numerous species of small RNA, including miRNAs, rRNAs, tRNAs and piRNAs, identified using bioinformatic tools after sequencing. A total of 996 and 979 miRNAs were identified in the MS patient and HC samples, respectively. Moreover, significant differences were observed between the miRNA expression profiles of the MS patients and the HC (Figure 1B).

To further narrow down the list of peripheral blood CD4^+^ T cell miRNAs, we identified miRNAs with > 200 sequencing reads and that exhibited a > 2-fold change in expression between the two groups. Ultimately, 15 miRNAs met these criteria (Supplementary Table 1), with 6 being significantly upregulated (fold-change > 2) and 9 being significantly downregulated (fold-change < 0.5) in the MS patients compared with their levels in the controls (Figure 1C). We selected the top four downregulated miRNAs (miR-150-5p, miR-423-5p, let-7e-5p and let-7f-5p) for further analysis.

### Downregulation of let-7f-5p expression in peripheral blood CD4^+^ T cells from MS patients and during the process of Th17 differentiation

**Figure 2:**
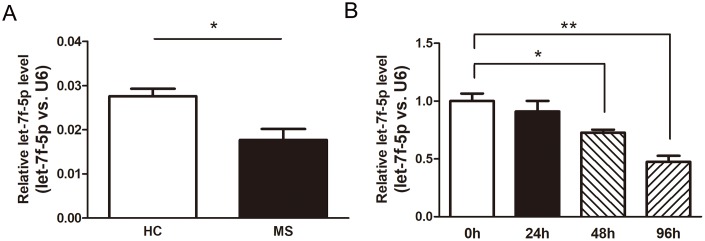
**Let-7f-5p expression is decreased during MS and downregulated in Th17 cells.** (**A**) CD4^+^ T cells were sorted from whole blood of multiple sclerosis (MS) patients (n = 6) and healthy controls (HC) (n = 6). The expression (let-7f-5p vs. U6) of let-7f-5p was analyzed by RT-PCR. (**B**) Naive CD4^+^ T cells were sorted from mouse spleens and maintained in Th17 differentiation medium. After 24, 48 and 96 h, the expression (let-7f-5p vs. U6) of let-7f-5p was analyzed by RT-PCR. (* *p* < 0.05, ** *p* < 0.01)

We performed real-time PCR (RT-PCR) assays using the peripheral blood CD4^+^ T cells from the MS patients (n = 6) and HC (n = 6) to confirm the results of the Illumina high-throughput sequencing assay (Supplementary Figure 1). The expression of let-7f-5p was significantly decreased in the MS patients compared with that observed in the HC (Figure 2A). In addition, naive CD4^+^ T cells were sorted from mouse spleens and maintained in Th17 differentiation medium, with the expression of let-7f-5p subsequently analyzed by RT-PCR. The results of this experiment revealed that let-7f-5p expression was markedly decreased during the process of Th17 differentiation (Figure 2B). These results suggested that let-7f-5p may play a role in Th17 differentiation.

### Let-7f-5p suppresses Th17 differentiation

**Figure 3:**
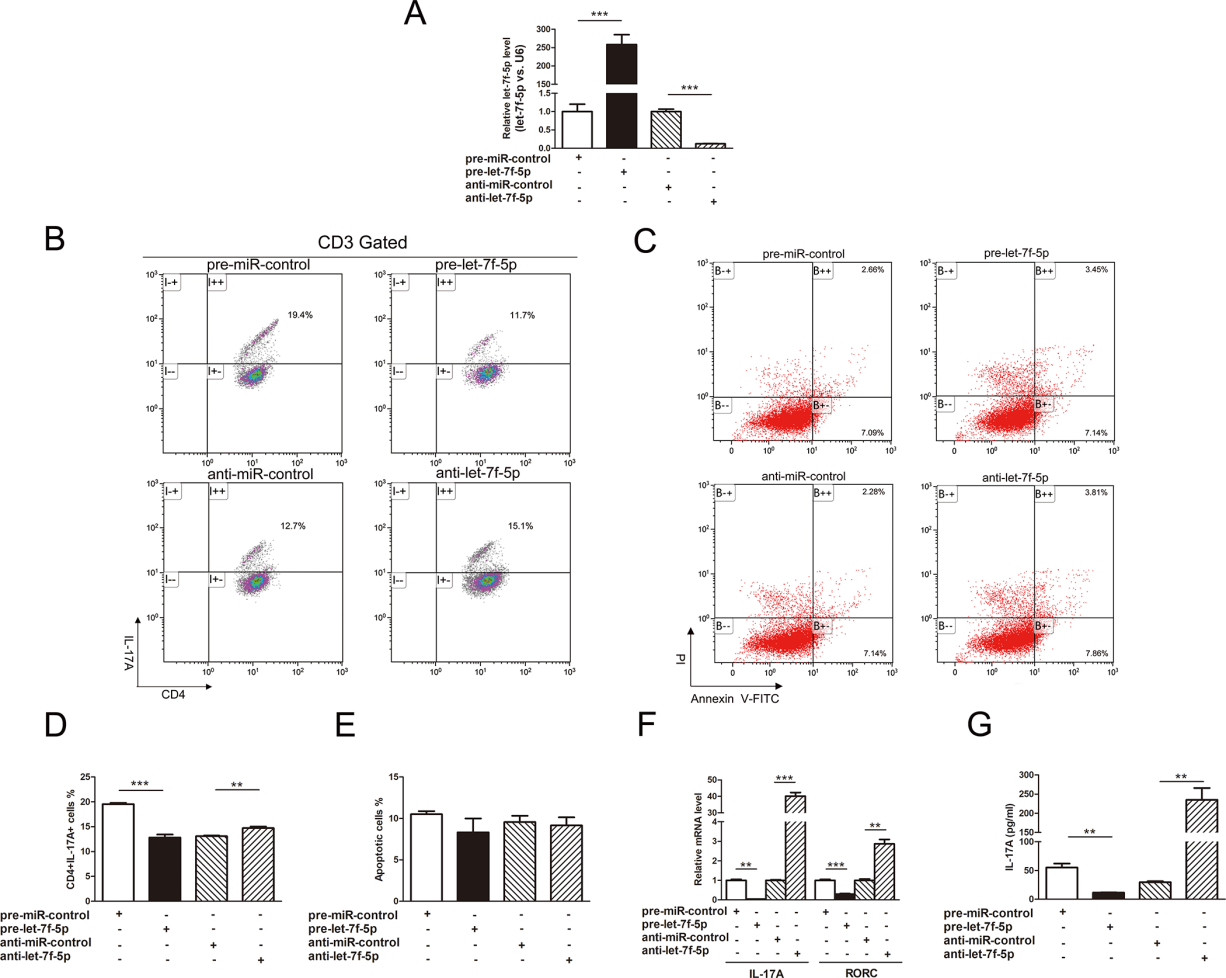
**Let-7f-5p suppresses Th17 differentiation.** (**A**) RT-PCR analysis of the expression of let-7f-5p in induced mouse Th17 cells transfected with 100 pmol of let-7f-5p mimic (pre-let-7f-5p), let-7f-5p inhibitor (anti-let-7f-5p) or negative control RNA (pre-miR-control or anti-miR-control). (**B**) The effect of let-7f-5p on Th17 differentiation. Naive CD4^+^ T cells were sorted from C57BL/6J mice, maintained in Th17 differentiation medium and transfected with pre-let-7f-5p, anti-let-7f-5p or negative control RNA for 96 h. The frequency of CD3^+^CD4^+^IL17A^+^ T cells is shown. The statistical analysis of the percentage of CD3^+^CD4^+^IL17A^+^ T cells is shown in (**D**). Data represent means ± SD from three independent experiments. (**C**, **E**) The effect of let-7f-5p on Th17 apoptosis. Induced mouse Th17 cells were cotransfected with pre-let-7f-5p, anti-let-7f-5p or negative control RNA. Cells were collected after 96 h and analyzed by flow cytometry. (**F**) Th17-specific gene expression in the transfected cells in (**A**) was analyzed by RT-PCR, and presented relative to GAPDH expression. (**G**) IL-17A expression in the culture supernatants of transfected cells in (**A**) was analyzed by ELISA. (* *p* < 0.05; ** *p* < 0.01; *** *p* < 0.001)

To explore the role of let-7f-5p in the process of Th17 differentiation, induced mouse Th17 cells were transfected with pre-let-7f-5p, anti-let-7f-5p or negative control RNA, and the let-7f-5p ectopic expression was verified by RT-PCR (Figure 3A). We subsequently observed that the frequency of CD3^+^CD4^+^IL17A^+^ T cells was significantly reduced in the pre-let-7f-5p-transfected cell population (Figure 3B, 3D). Furthermore, Th17-specific genes, IL-17A and RAR related orphan receptor C (RORC), and the concentration of IL-17A in the cell culture supernatants of these cells were significantly decreased as well (Figure 3F, 3G). In contrast, the frequency of CD3^+^CD4^+^IL17A^+^ T cells, Th17-specific gene expression and the concentration of IL-17A in the cell culture supernatants were elevated in the inhibitor-transfected cell population (Figure 3). In addition, no obvious differences in apoptosis were observed among the induced mouse Th17 cells transfected with pre-let-7f-5p, anti-let-7f-5p or negative control RNA (Figure 3C, 3E). These results demonstrated that let-7f-5p suppresses Th17 cell differentiation without impacting apoptosis.

### Prediction of STAT3 as a target gene of let-7f-5p

**Figure 4:**
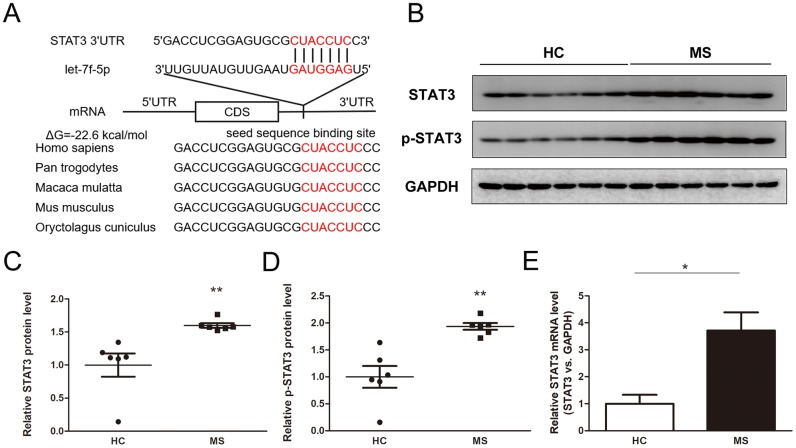
**Identification of STAT3 as a direct target gene of let-7f-5p.** (**A**) Sequence pairing between mature let-7f-5p and the human STAT3 3’-UTR. All of the nucleotides of the seed sequence for the binding site are conserved in several species, including *Homo sapiens, Pan trogodytes, Macaca mulatta, Mus musculus, Oryctolagus cuniculus*. The predicted free energy values of the hybrids are indicated. (**B**) Western blot analysis of STAT3 and p-STAT3 protein expression levels in CD4^+^ T cells from MS patients (n = 6) and HC (n = 6); (**B**) representative images; (**C**–**D**) quantitative analysis of STAT3 and p-STAT3 protein level. (**E**) RT-PCR analysis of STAT3 mRNA levels in CD4^+^ T cells from MS patients and HC. (* *p* < 0.05; ** *p* < 0.01; *** *p* < 0.001)

We used TargetScan, miRanda and PicTar to predict the potential target genes of let-7f-5p to explore the molecular mechanisms of let-7f-5p-mediated regulation of Th17 cell differentiation. STAT3 was identified as a target regulated by let-7f-5p because it was predicted to be targeted by let-7f-5p has an important role in Th17 differentiation. The predicted binding between let-7f-5p and the STAT3 3’-UTR is presented in Figure 4A, which shows that one highly conserved let-7f-5p binding site was identified in the 3’-UTR of the STAT3 mRNA transcript. The minimum free energy value of hybridization was -22.6 kcal/mol, which is within the range of genuine miRNA-mRNA pairs. Therefore, STAT3 was selected for further analysis.

### Detection of inverse correlations between let-7f-5p and STAT3/p-STAT3 protein levels in peripheral blood CD4^+^ T cells from MS patients

The levels of STAT3 and p-STAT3 were detected in the peripheral blood CD4^+^ T cells from the MS patients and HC, the results of which showed that the levels of both were dramatically increased in the MS patient samples compared with those from the HC (Figure 4B–4D). In addition, the expression of STAT3 at the mRNA level was significantly upregulated in the MS group compared with that in the HC (Figure 4E). Furthermore, the expression of let-7f-5p was inversely correlated with the levels of STAT3 and p-STAT3 (Supplementary Figure 2).

### Validation of STAT3 as a direct target of let-7f-5p

**Figure 5:**
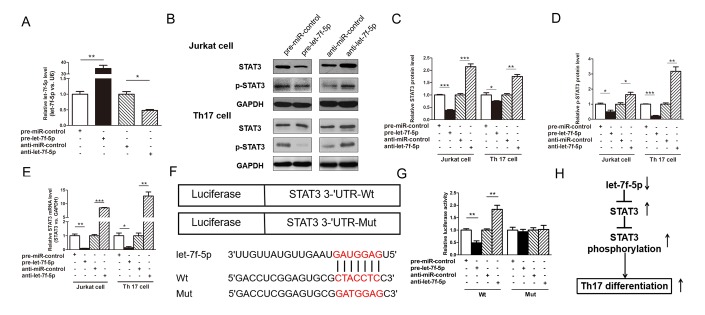
**STAT3 is a direct target of let-7f-5p.** (**A**) RT-PCR analysis of the expression of let-7f-5p in Jurkat cells transfected with 100 pmol of pre-let-7f-5p, anti-let-7f-5p or negative control RNA. (**B**) Western blotting analysis to detect STAT3 and p-STAT3 protein levels in Jurkat cells and induced mouse Th17 cells (Th17 cell) transfected with 100 pmol of the pre-let-7f-5p, anti-let-7f-5p or negative control RNA. (**B**): representative image; (**C**, **D**) quantitative analysis. (**E**) RT-PCR analysis of STAT3 mRNA levels in Jurkat cells and induced mouse Th17 cells transfected with 100 pmol of the pre-let-7f-5p, anti-let-7f-5p or negative control RNA. (**F**) Luciferase plasmids contain the wild-type (Wt) and mutant 3’-UTR of STAT3 and sequences of Wt and Mut target sites for let-7f-5p in the 3’-UTR of STAT3 are shown. (**G**) Dual luciferase reporter assay was used to confirm the direct recognition of the STAT3 3’-UTR by let-7f-5p. Wt and Mut luciferase plasmids were cotransfected into 293T cells with 100 pmol of pre-let-7f-5p, anti-let-7f-5p or negative control RNA. The β-galactosidase (β-gal) expression plasmid was used as a transfection control. (**H**) The working model of the role of let-7f-5p in Th17 differentiation. (* *p* < 0.05; ** *p* < 0.01; *** *p* < 0.001)

The ability of let-7f-5p to regulate STAT3 was evaluated by assessing STAT3 expression in Jurkat cells after the overexpression or knockdown of let-7f-5p. In these experiments, Jurkat cells were transfected with pre-let-7f-5p or anti-let-7f-5p to overexpress or knockdown let-7f-5p expression, respectively. As shown in Figure 5A, let-7f-5p was successfully overexpressed and knocked down in Jurkat cells. The protein levels of STAT3 and p-STAT3 were significantly decreased by the overexpression of let-7f-5p, whereas the knockdown of let-7f-5p expression significantly increased the levels of STAT3 and p-STAT3 protein in Jurkat cells (Figure 5B–5D). To determine the level at which let-7f-5p affects STAT3 expression, we assessed STAT3 mRNA expression in let-7f-5p-overexpressing or let-7f-5p-knockdown Jurkat cells. Both the overexpression and knockdown of let-7f-5p influenced STAT3 mRNA levels, indicating that let-7f-5p regulates the STAT3 protein level at the transcriptional level (Figure 5E). To confirm the results of these experiments, we repeated the above experiments in induced mouse Th17 cells and observed consistent results (Figure 5A–5E).

To explore whether the negative regulatory effects of let-7f-5p on STAT3 were mediated by the binding of let-7f-5p to the predicted site in the 3’-UTR of STAT3, we used a luciferase reporter plasmid harboring the STAT3 3’-UTR sequence. The plasmid was cotransfected into 293T cells with the pre-let-7f-5p, anti-let-7f-5p or negative control RNA. As anticipated, compared with pre-miR-control-transfected cells, those transfected with pre-let-7f-5p exhibited a nearly 50% reduction in luciferase activity. In contrast, compared with cells transfected with the anti-miR-control, those transfected with anti-let-7f-5p exhibited an increase in luciferase activity (Figure 5F, 5G). Subsequently, we mutated the corresponding complementary site in the STAT3 3’-UTR to eliminate the predicted let-7f-5p binding site. The luciferase activity observed using the mutant remained unchanged regardless of how let-7f-5p expression was altered (Figure 5F, 5G). These results demonstrate that the predicted let-7f-5p binding site in the STAT3 3’-UTR contributes to the miRNA-mRNA binding.

Recent years, several studies have demonstrated that let-7 family miRNAs can directly target IL-6 [20–22], the activator of STAT3 signaling, so we examined whether the role of let-7f-5p on STAT3 expression and phosphorylation are specific to IL-6 signaling. As we know, IL6, IL-21 and IL-23 are all activators of STAT3 expression and phosphorylation [23]. To confirm whether the effects of let-7f-5p on STAT3 expression and phosphorylation are specific to IL-6 signaling, we added IL-21 and IL-23, respectively, instead of IL-6 at the beginning of Th17 differentiation. As shown in Supplementary Figure 3, these results indicated that the role of let-7f-5p on STAT3 expression and phosphorylation is not specific to IL-6. In summary, the above finding suggests that let-7f-5p directly recognizes and binds to the 3’-UTR of the STAT3 mRNA transcript, resulting in mRNA degradation.

## DISCUSSION

In this study, we found a new Th17 cell-associated miRNA, let-7f-5p, which exhibits downregulated expression in peripheral blood CD4^+^ T cells from MS patients and in the process of Th17 differentiation. We also demonstrated that let-7f-5p suppresses Th17 differentiation in the pathogenesis of MS by targeting STAT3, a working model for which is shown in Figure 5H. This study uncovered the biological mechanism of let-7f-5p in the differentiation of Th17 cells and in the pathogenesis of MS.

MS is a chronic demyelinating neurodegenerative autoimmune disease of the CNS that affects nearly 2.5 million people, with a prevalence of 30 cases per 100,000 people [24]. Progress has been made towards understanding the pathogenesis of MS, but its precise mechanism remains largely unelucidated. Thus, there is tremendous need to identify susceptibility factors and new therapeutic targets for MS.

In the past decade, a class of small, conserved single-stranded noncoding RNA molecules called miRNAs has emerged as crucial factors in autoimmune diseases, such as rheumatoid arthritis (RA), inflammatory bowel disease, systemic lupus erythematosus (SLE), myasthenia gravis and MS [25–27]. Several unique miRNAs have been shown to function in the pathogenesis of MS and EAE through different Th17-related pathways by targeting specific proteins. For example, miR-326 is reported as a Th17 cell–associated miRNA and promotes Th17 differentiation by targeting Ets-1, a negative regulator of Th17 differentiation [28]. MiR-15b is a key regulator in the pathogenesis of EAE that suppresses Th17 differentiation by targeting O-GlcNAc transferase (OGT) [29]. More severe EAE is observed in miR-146a^-/-^ mice than in wild-type mice as a result of changes in the regulation of Th17 differentiation [30]. Moreover, many miRNAs have been shown to have significantly upregulated or downregulated expression in the peripheral blood, peripheral blood mononuclear cells (PBMCs), CSF and brain lesions of MS patients [31–34]. To explore the miRNA expression profile of peripheral blood CD4^+^ T cells from MS patients, we performed a high-throughput sequencing analysis to identify miRNAs with significantly altered expression levels between samples from MS patients and those from HC. The results showed that miR-150-5p, miR-423-5p, let-7e-5p and let-7f-5p had markedly downregulated expression in the MS group. To validate the results of the Illumina high-throughput sequencing analysis, we performed RT-PCR in a cohort of MS patients and HC, and showed that the expression of let-7f-5p was downregulated in CD4^+^ T cells from MS patients, whereas the expression of miR-150-5p, miR-423-5p, let-7e-5p remained unchanged. In addition, the expression of let-7f-5p was further found to have a remarkable decrease during the process of Th17 differentiation.

The let-7 family is miRNA family that is highly conserved in sequence and function across species. Let-7 was originally detected in the nematode *Caenorhabditis elegans* as one of the first identified miRNAs [35], and fourteen members of this family have been subsequently recognized [36]. Let-7f-5p is located in the intergenic region at 9q22.3 and is reported to be involved in a series of physiological and pathological processes, including angiogenesis [37], immune response [38], immunocyte differentiation [39] and microbial infection [40]. As having been reported, let-7f-5p expression was significantly downregulated in the peripheral blood of MS patients [41–43]. In addition, it has been shown that let-7f-5p inhibits IL-23R expression in human CD4^+^ memory T cells [44]. In this study, we shown that let-7f-5p can suppress the Th17 differentiation without influencing apoptosis, leading to the decreased IL-17A secretion.

To illuminate the let-7f-5p-disrupted pathway involved in the pathogenesis of MS, potential let-7f-5p targets were predicted and identified. We identified STAT3 as a putative let-7f-5p target gene. STAT3 is a key Th17 differentiation-specific signaling protein [45], which is required for commitment of naive CD4^+^ T cells to the Th17 developmental pathway [46]. As IL-6, TGF-β, IL-21 and IL-23 are involved in Th17 differentiation, stimulation with these molecules triggers Janus kinase 2 (JAK-2) activation and the subsequent phosphorylation of STAT3 [23]. STAT3 can bind to the promoter of the RORC gene and STAT3-deficient T cells have impaired the expression of RORγt [47], which is the master regulator of Th17 differentiation [48]. The overexpression of STAT3 is sufficient to induce Th17 cells and the secretion of IL-17 [46, 49], while the deletion of STAT3 abrogates Th17 differentiation and IL-17 secretion [50]. Mice with targeted deletion of STAT3 in the CD4^+^ T cell are completely resistant to EAE [50]. Furthermore, it has been reported that the expression level of STAT3 and p-STAT3 are significantly higher in PBMCs from MS patients during relapses compared with remission [51, 52]. Thus, we showed that the expression of both STAT3 and p-STAT3 was significantly elevated in the peripheral blood CD4^+^ T cells from the MS patients and confirmed that the downregulation of let-7f-5p expression resulted in the upregulation of STAT3 expression and phosphorylation, which in turn, enhanced the differentiation of Th17 cells. This inverse relationship was confirmed by monitoring the effect of the up- and downregulation of let-7f-5p expression on STAT3 and p-STAT3 protein levels in Jurkat and induced mouse Th17 cells. We also demonstrated that let-7f-5p downregulated the expression of STAT3 at the transcriptional level, showing that let-7f-5p targeting of STAT3 mRNA inhibits the differentiation of Th17 cells. Furthermore, we revealed that the role of let-7f-5p on STAT3 expression and phosphorylation was not specific to IL-6. This finding indicates that STAT3 acts as a downstream regulator of let-7f-5p in Th17 differentiation and pathogenesis of MS.

In conclusion, the present study not only revealed the critical role of let-7f-5p in the pathogenesis of MS but also explored the biological mechanism through which let-7f-5p suppressed Th17 differentiation and identified STAT3 as a direct target. This study may provide insight into the pathogenesis of MS and supply a new therapeutic target.

## MATERIALS AND METHODS

### Patients and control samples

Prior to this study, all of the patients and volunteers provided written informed consent. The study was conducted in accordance with the Declaration of Helsinki, and the protocol was approved by the Ethical Committee of Harbin Medical University. The clinical information for the MS patients and HC including age, sex, the percentage of brain magnetic resonance imaging (MRI) abnormalities and Expanded Disability Status Scale (EDSS) scores is shown in Table 1. Whole peripheral blood samples (with EDTA) were drawn from treatment-naive relapsing-remitting multiple sclerosis (RRMS) patients at relapse (n = 16) and from age/gender-matched healthy volunteers (n = 16) attending the Second Affiliated Hospital of Harbin Medical University between 2014 and 2017. MS patients were diagnosed based on the McDonald criteria (2010) [3]. Patients who had a history of cancer, other CNS diseases, or additional autoimmune diseases were excluded.

**Table 1:** Characteristics of MS patients and HC.

	**HC**	**MS**	***p*-value**
Sample size, n	16	16	-
Age, mean ± SD	35.06 ± 8.54	38.56 ± 11.54	0.35^a^
Sex, n			1^b^
Male	4	4	
Female	12	12	
Brain MRI abnormalities (%)	NA	16/16 (100%)	-
EDSS score, mean ± SD	NA	3.23 ± 0.59	-

HC, healthy controls; MS, multiple sclerosis; MRI, magnetic resonance imaging; EDSS, expanded disability status scale; SD, standard deviation; NA, not applicable; a, Student’s-t test; b, Two-sided χ^2^ test.

### CD4^+^ T cell separation

A two-step process was performed to separate CD4^+^ T cells from whole blood. In the first step, lymphocyte separation medium was used to isolate the PBMCs following the manufacturer’s protocols. In the second step, CD4^+^ T cells were separated from the PBMCs via flow cytometric sorting (BD Biosciences, USA). The purity of the CD4^+^ T cells as measured by flow cytometry (FCM) (BD Biosciences) was > 90% (Supplementary Figure 4).

### Mice

Female C57BL/6J mice (6-8 weeks old) were purchased from the Model Animal Research Center of Nanjing University (Nanjing, China). All animals were maintained under specific pathogen-free conditions at Nanjing University and all of the animal procedures were approved by the Institutional Animal Care and Research Advisory Committee of Nanjing University.

### Cell culture

Jurkat and human embryonic kidney (HEK) 293T cells were purchased from the Shanghai Institute of Cell Biology of the Chinese Academy of Sciences (Shanghai, China). Jurkat cells were plated in flasks containing RPMI 1640 medium (Gibco, Carlsbad, CA, USA) supplemented with 10% fetal bovine serum (FBS, Gibco) and 1% penicillin/streptomycin (PS) and incubated in a humidified chamber (37°C, 5% CO_2_). HEK293T cells were cultured in DMEM supplemented with 10% FBS and 1% PS in a humidified chamber (37°C, 5% CO_2_).

### Naive CD4^+^ T cell separation and differentiation

Naive CD4^+^ T cells were separated by sorting CD4^+^CD62L^+^ cells from the spleens of female C57BL/6J mice with a mouse naive CD4^+^ T cell isolation kit (Biolegend, CA, USA). The purity of the sorted naive mouse CD4^+^ T cells was confirmed by FCM with an anti-mouse CD4 FITC-conjugated antibody (eBioscience, CA, USA) and an anti-mouse CD62L PE-conjugated antibody (eBioscience). For Th17 differentiation, the naive CD4^+^ T cells were activated with 1 μg/mL anti-CD3 antibody (eBioscience) and 1 μg/mL anti-CD28 antibody (eBioscience) for 36 h and were subsequently maintained for 96 h under Th17 cell-polarizing conditions: RPMI 1640 medium supplemented with 10% FBS, 2.5 ng/mL TGF-β (R&D Systems), 20 ng/mL IL-6 (R&D Systems, MN, USA), 10 μg/mL anti–IL-4 antibody (eBioscience) and 10 μg/mL anti–IFN-γ antibody (eBioscience).

### RT-PCR

Total RNA was extracted from cells using TRIzol reagent (Invitrogen, MA, USA). Subsequently, the RNA was reverse transcribed into cDNA with avian myeloblastosis virus (AMV) reverse transcriptase (TaKaRa, Dalian, China) and a stem-loop RT primer (Applied Biosystems, Foster City, CA). RT-PCR was performed on the Applied Biosystems 7500 Sequence Detection System using TaqMan miRNA probes (Applied Biosystems). The relative expression level of let-7f-5p was normalized to the level of U6 using the equation 2^−ΔΔCT^, where ΔΔCT = (CT_let-7f-5p_ − CT_U6_) _target_ − (CT_let-7f-5p_ − CT_U6_) _control_.

To assess mRNA expression, total RNA was reverse transcribed into cDNA using oligo d(T)18 primers (TaKaRa). Next, RT-PCR was performed using SYBR Green and specific primers, the detailed sequences of which are shown in Supplementary Table 2. The relative mRNA expression level of each gene of interest was normalized to the mRNA level of GAPDH using a method similar to the one described above.

### Illumina high-throughput sequencing

Illumina high-throughput sequencing of RNA samples was performed by BGI (Wuhan, China). Briefly, following the filtration of small RNAs (< 30 bases) using a PAGE gel, adaptors were ligated to the 5’ and 3’ ends of the small RNAs. The small RNAs were then amplified through 17 cycles using adaptor-specific primers. The fragments were subsequently purified from an agarose gel, and the purified DNA was used to perform high-throughput sequencing following the manufacturer’s recommendations.

### Protein extraction and Western blotting

The proteins were extracted from the samples using RIPA lysis buffer (Beyotime, Shanghai, China) supplemented with a phosphatase inhibitor (Thermo Fisher Scientific, Rockford, Cambridge, MA) and were separated on a 10% SDS-PAGE gel. Western blotting was performed using anti-STAT3 and anti-p-STAT3 antibodies (Cell Signaling Technology, #9132 and #9131, respectively) as well as an anti-GAPDH antibody (sc-365062, Santa Cruz, CA, USA). The protein bands were detected using FLICapture (Tanon, Shanghai, China), and the images were analyzed using ImageJ 1.50i.

### Overexpression or knockdown of let-7f-5p

The overexpression or knockdown of let-7f-5p was achieved using let-7f-5p mimics or inhibitors, respectively, in Jurkat cells and induced mouse Th17 cells. A synthetic let-7f-5p mimic (pre-let-7f-5p), an inhibitor (anti-let-7f-5p) and negative control RNAs (pre-miR-control and anti-miR-control) (RiboBio, Guangzhou, China) were used. For transfection, Lipofectamine 2000 (Invitrogen) was used in procedures following the manufacturer’s instructions. Moreover, anti-CD3 and anti-CD28 antibodies were used to activate the naive CD4^+^ T cells for 36 h and after which the cells were transfected with RNA oligoribonucleotides using a BTX™ Gemini X2 Electroporation System (BTX Harvard Apparatus, Holliston, MA, USA), with pulse parameters of 500 volts and a 0.8 ms duration. For each transfection, 100 pmol of pre-let-7f-5p, anti-let-7f-5p or negative control RNA was used in each transfection.

### Luciferase reporter assay

To explore whether let-7f-5p directly regulates STAT3 expression, the STAT3 3’-UTR sequence was inserted into a luciferase reporter plasmid. A STAT3 3′-UTR with the sequence mutated from TGAATGT to ACTTACA was cloned into an identical luciferase vector to construct the plasmid STAT3-Mut (GenScript, Nanjing, China). HEK293T cells were cotransfected with 1 μg of luciferase reporter plasmid, 1 μg of β-galactosidase (β-gal) expression plasmid and 100 pmol of pre-let-7f-5p, anti-let-7f-5p or negative control RNA. The β-gal expression plasmid was used as a transfection control. Luciferase activity was measured with a luciferase assay kit (Promega, Madison, WI, USA) using a Modulus Luminometer (Turner Biosystems, Sunnyvale, USA).

### ELISA for IL-17A expression

The concentration of IL-17A in the cell-cultured supernatant from the induced mouse Th17 cells was measured using a mouse IL-17A enzyme-linked immunosorbent assay (ELISA) kit (R&D Systems) according to the manufacturer’s recommendations.

### Cell apoptosis assay

Induced mouse Th17 cells were cotransfected with pre-let-7f-5p, anti-let-7f-5p or negative control RNA. After 96 h, cells were analyzed using an Apoptosis Detection Kit (BD Biosciences) by FCM (Backman Coulter, USA) within 1 h of staining.

### Intracellular staining and flow cytometry

Induced mouse Th17 cells were cotransfected with pre-let-7f-5p, anti-let-7f-5p or negative control RNA. Subsequently, a mouse Th17 staining kit (MULTI SCIENCES, China) was used to analyze the cells after 96 h by FCM (Beckman Coulter, USA) following the manufacturer’s recommendations.

### Statistical analysis

All of the data are shown as the means ± SD. Statistical analyses were conducted using Student’s t-test and *p* < 0.05 was considered to be significant. Prism 5.0 (GraphPad, USA) was used to perform statistical analyses.

## Supplementary Material

Supplementary Figures

Supplementary Tables
