# Supplementary material for: Let-7f-5p suppresses Th17 differentiation via targeting STAT3 in multiple sclerosis
Source: Aging (Albany NY). 2019 Jul 15;11(13):4463–77. doi: 10.18632/aging.102093 (PMC6660039; doi:10.18632/aging.102093)
Supplement: Supplementary Figures [file aging-11-102093-s002.pdf]

## SUPPLEMENTARY FIGURES

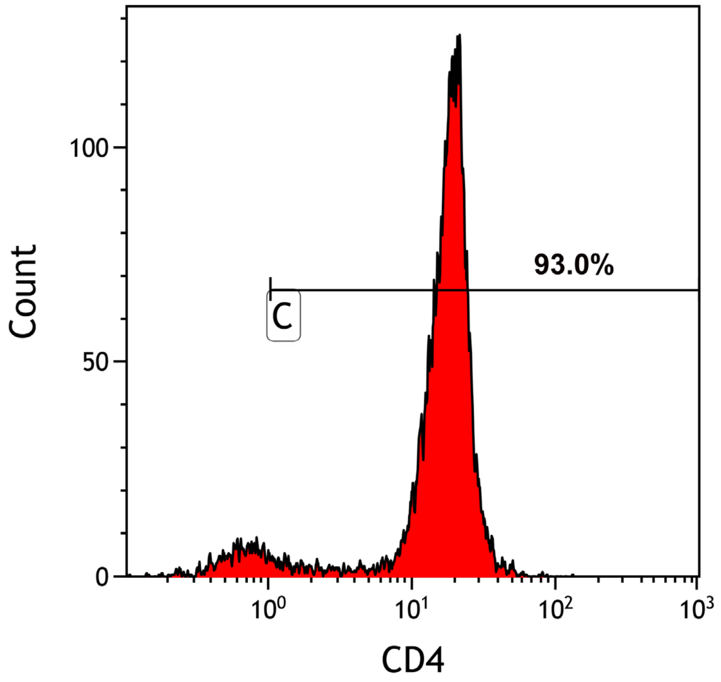


**Supplementary Figure 1. The expression of miR-150-5p, miR-423-5p, let-7e-5p of peripheral blood CD4$^+$ T cells from MS patients and HC.**

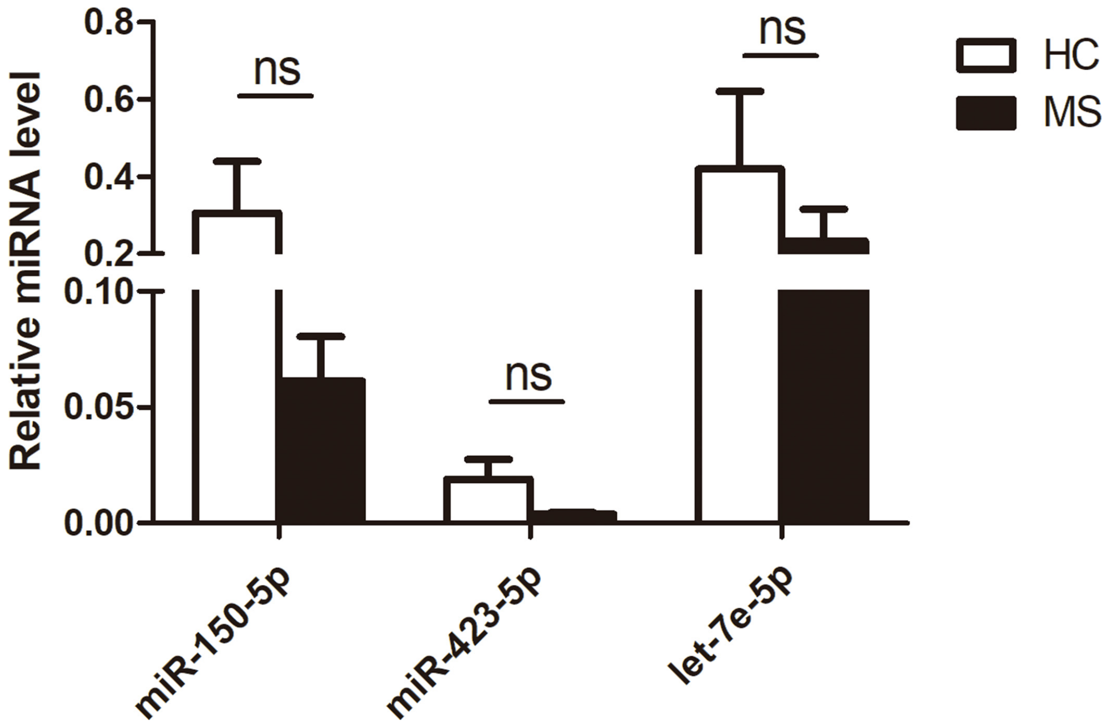


**Supplementary Figure 2. The inverse correlations between let-7f-5p and STAT3/p-STAT3 protein levels.**

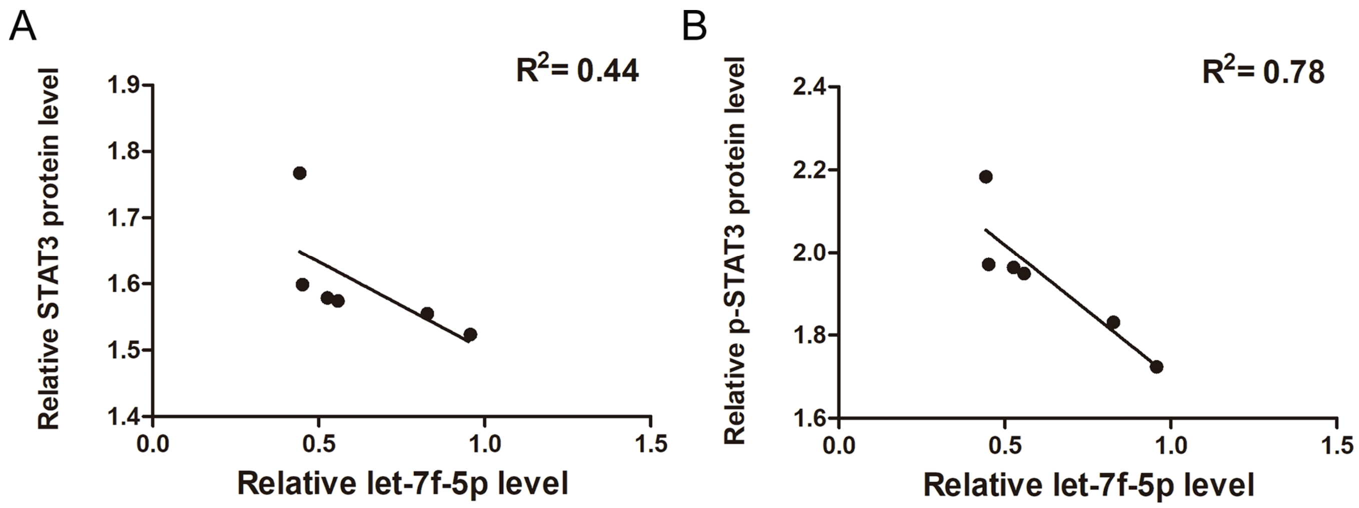


**Supplementary Figure 3. The role of let-7f-5p on STAT3 expression and phosphorylation is not specific to IL-6.**

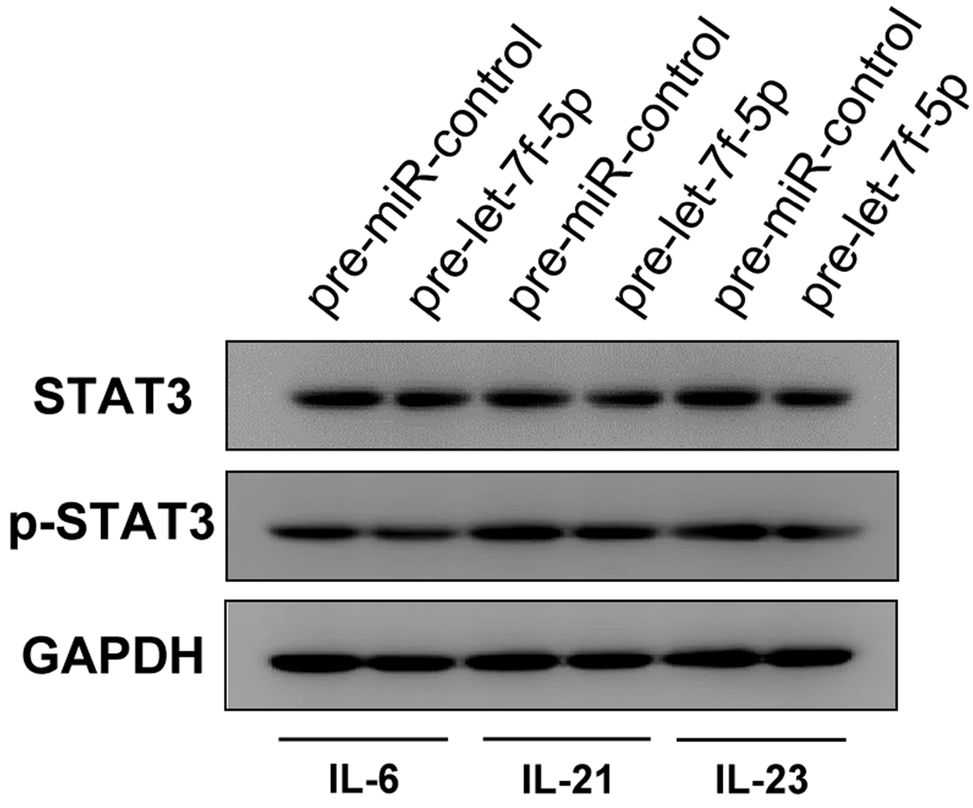


**Supplementary Figure 4. Purity of the sorted $CD4^+$ T cells.**
