# Supplementary material for: Let-7f-5p suppresses Th17 differentiation via targeting STAT3 in multiple sclerosis
Source: Aging (Albany NY). 2019 Jul 15;11(13):4463–77. doi: 10.18632/aging.102093 (PMC6660039; doi:10.18632/aging.102093)
Supplement: Supplementary Tables [file aging-11-102093-s001.pdf]

SUPPLEMENTARY TABLES

Supplementary Table 1. miRNAs differentially expressed in CD4+ T cells from MS patients.

miRNA	Copy number in healthy control	Copy number in MS patients	Fold change
hsa-miR-150-5p	103530	20832	0.176816017
hsa-miR-423-5p	102714	20992	0.179589533
hsa-let-7e-5p	5990	1527	0.224010875
hsa-let-7f-5p	914225	283457	0.272452591
hsa-miR-744-5p	8685	3084	0.312033669
hsa-miR-423-3p	33469	12411	0.32585237
hsa-miR-222-3p	23968	9097	0.333521044
hsa-miR-181a-2-3p	4318	1882	0.382995647
hsa-let-7d-5p	10819	4736	0.384663889
hsa-let-7b-5p	1885	4801	2.238088414
hsa-miR-26b-5p	24774	63399	2.248760097
hsa-miR-221-3p	9269	28492	2.701138752
hsa-miR-451a	11659	39549	2.980787735
hsa-miR-19b-3p	6163	29161	4.157833396
hsa-miR-19a-3p	1987	10293	4.551986374

Supplementary Table 2. The primers used for real-time PCR.

Name		Sequences
**Human**		
STAT3	Forward primer (5′>3′)	CAGCAGCTTGACACACGGTA
	Reverse primer (5′>3′)	AAACACCAAAGTGGCATGTGA
RORC	Forward primer (5′>3′)	GTGGGGACAAGTCGTCTGG
	Reverse primer (5′>3′)	AGTGCTGGCATCGGTTTCG
IL-17A	Forward primer (5′>3′)	TCCCACGAAATCCAGGATGC
	Reverse primer (5′>3′)	GGATGTTCAGGTTGACCATCAC
GAPDH	Forward primer (5′>3′)	CGAGCCACATCGCTCAGACA
	Reverse primer (5′>3′)	GTGGTGAAGACGCCAGTGGA
**Mouse**		
STAT3	Forward primer (5′>3′)	CAATACCATTGACCTGCCGAT
	Reverse primer (5′>3′)	GAGCGACTCAAACTGCCCT
RORC	Forward primer (5′>3′)	AGTGTAATGTGGCCTACTCCT
	Reverse primer (5′>3′)	GCTGCTGTTGCAGTTGTTTCT
IL-17A	Forward primer (5′>3′)	TTTAACTCCCTTGGCGCAAAA
	Reverse primer (5′>3′)	CTTTCCCTCCGCATTGACAC
GAPDH	Forward primer (5′>3′)	AGGTCGGTGTGAACGGATTTG
	Reverse primer (5′>3′)	TGTAGACCATGTAGTTGAGGTCA
